# Beta-hemolytic *Streptococcus dysgalactiae* strains isolated from horses are a genetically distinct population within the *Streptococcus dysgalactiae* taxon

**DOI:** 10.1038/srep31736

**Published:** 2016-08-17

**Authors:** Marcos D. Pinho, Erdal Erol, Bruno Ribeiro-Gonçalves, Catarina I. Mendes, João A. Carriço, Sandra C. Matos, Silvia Preziuso, Antina Luebke-Becker, Lothar H. Wieler, Jose Melo-Cristino, Mario Ramirez

**Affiliations:** 1 Instituto de Microbiologia, Instituto de Medicina Molecular, Faculdade de Medicina, Universidade de Lisboa, Lisbon, Portugal; 2 Department of Veterinary Science, Veterinary Diagnostic Laboratory, University of Kentucky, Lexington, Kentucky, United States; 3 Department of Veterinary Medical Sciences, University of Camerino, Matelica, Italy; 4 Institute of Microbiology and Epizootics, Freie Universität Berlin, Germany; 5 Robert Koch-Institute, Berlin, Germany

## Abstract

The pathogenic role of beta-hemolytic *Streptococcus dysgalactiae* in the equine host is increasingly recognized. A collection of 108 Lancefield group C (*n* = 96) or L (*n* = 12) horse isolates recovered in the United States and in three European countries presented multilocus sequence typing (MLST) alleles, sequence types and *emm* types (only 56% of the isolates could be *emm* typed) that were, with few exceptions, distinct from those previously found in human *Streptococcus dysgalactiae* subsp. *equisimilis*. Characterization of a subset of horse isolates by multilocus sequence analysis (MLSA) and 16S rRNA gene sequence showed that most equine isolates could also be differentiated from *S. dysgalactiae* strains from other animal species, supporting the existence of a horse specific genomovar. Draft genome information confirms the distinctiveness of the horse genomovar and indicates the presence of potentially horse-specific virulence factors. While this genomovar represents most of the isolates recovered from horses, a smaller MLST and MLSA defined sub-population seems to be able to cause infections in horses, other animals and humans, indicating that transmission between hosts of strains belonging to this group may occur.

*Streptococcus dysgalactiae* subsp. *equisimilis* (SDE) is a beta-hemolytic streptococcus which may present Lancefield groups C, G, L or A that is being increasingly reported in human infections^1^,^2^,^3^. In the human host it causes pharyngitis, skin and soft tissues infections, bacteremia and other life-threatening conditions, closely resembling the spectrum of infections caused by *Streptococcus pyogenes* (group A *Streptococcus* [GAS])^4^. The SDE epithet has also been commonly used to identify beta-hemolytic *S. dysgalactiae* strains isolated from a wide range of animal hosts, including companion animals, livestock (such as horses, pigs, sheep and cows) and wild animals^5^,^6^. In horses, these strains are believed to be part of the microbiota of the skin and mucosal surfaces^7^ and have been isolated from infections of the horse reproductive system, upper respiratory tract infections, including cases of strangles-like disease, and less frequently from other body sites^8^,^9^,^10^.

The beta-hemolytic *S. dysgalactiae* isolates from horses and other animals can be distinguished from human SDE by phenotypic and genotypic methods^11^,^12^. Differences in the streptokinase genes of human and horse isolates have been used to discriminate between these isolates^13^, and one study employing multilocus sequence analysis (MLSA)^12^ concluded that the beta-hemolytic animal isolates were more closely related to the non-hemolytic *Streptococcus dysgalactiae* subsp. *dysgalactiae* (SDD) strains from bovine mastitis than to human SDE. Although these observations suggest that human and horse *S. dysgalactiae* could constitute distinct populations, this has not been explored.

Molecular typing data has been accumulating for human SDE but is lacking for animal isolates. *emm* typing, relying on the sequence analysis of the *emm* gene encoding the M-protein present in SDE and GAS genomes, has been the most commonly used typing technique for human SDE^2^,^3^,^14^,^15^, but seldom applied in animal isolates. Thus, among the approximately 80 *emm* types currently known for SDE, only a few were derived from animal hosts, including horses (http://www.cdc.gov/streplab/M-ProteinGene-typing.html). Similarly, the multilocus sequence typing (MLST) scheme for *S. dysgalactiae* (http://pubmlst.org/sdysgalactiae/) has been used almost exclusively in SDE recovered from humans^15^,^16^,^17^, and while fourteen whole-genome sequences are available for this subspecies, a single genome is available from a bovine SDD isolate (http://www.ncbi.nlm.nih.gov/genome/823).

The aim of the current work was the genotypic characterization of a collection of *S. dysgalactiae* isolates recovered from horses, in order to clarify their genetic relationship to other *S. dysgalactiae* strains. The application of MLST and *emm* typing allowed the comparison with a large number of SDE previously described from human infections. A representative subset of horse isolates was chosen for MLSA, 16S rRNA gene sequencing and whole-genome analysis, allowing to define the specific genotypic characteristics of the isolates of equine origin and revealing a unique population in this host, distinct from other *S. dysgalactiae* associated with infection in humans and other animals.

## Results

### Characteristics of the horse isolates

All horse isolates were beta-hemolytic in sheep blood agar after overnight incubation. Most isolates presented the Lancefield group C antigen (*n* = 96), while 12 isolates, recovered in the United States (*n* = 10) and Italy (*n* = 2), had the Lancefield group L antigen.

### Clonality and MLST-based comparison with human SDE

**Figure 1:**
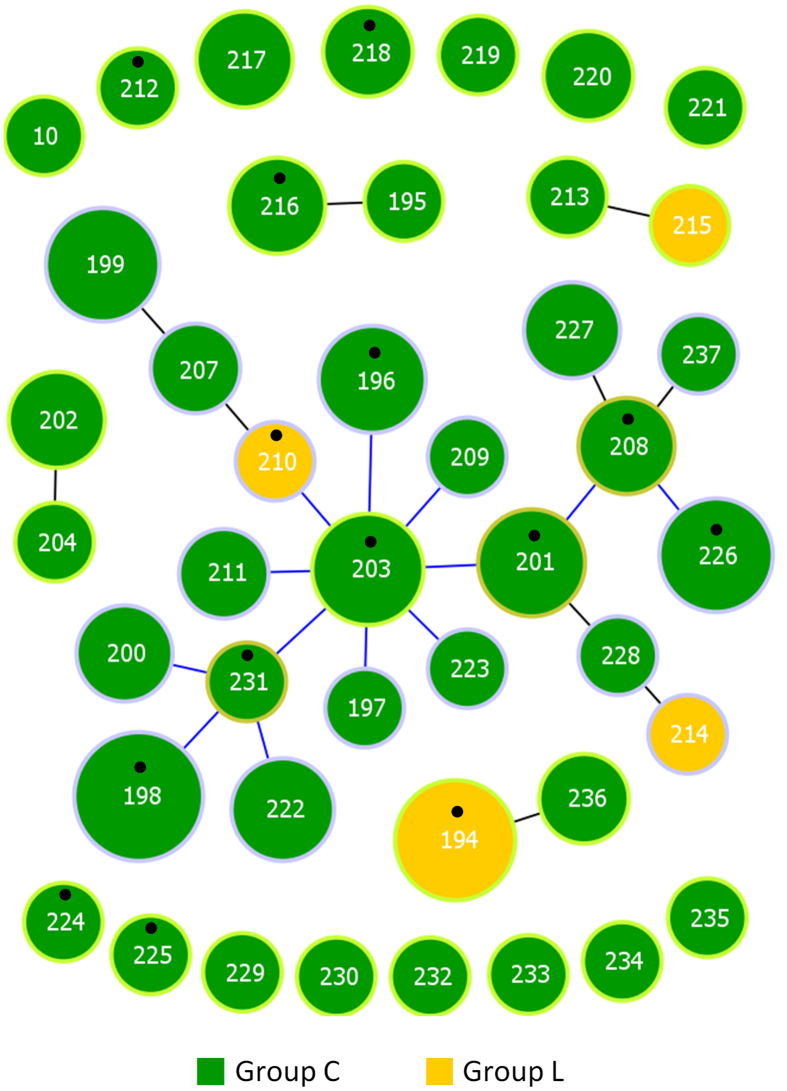
goeBURST diagram of 108 Lancefield group C and L horse isolates. The size of each circle is proportional to the number of isolates with that particular ST on a logarithmic scale. The number of isolates with the same characteristic is proportional to the respective color. STs assigned to the same CC at the SLV level are linked by straight lines. The line colors refer to the tiebreak level reached before choosing which link to draw according to the goeBURST algorithm implemented in PHYLOViZ. Putative CC founders are identified by a light green circle and correspond to the STs with the higher number of SLVs. Putative sub-founders are identified by a light brown circle and correspond to STs with at least three SLVs. STs corresponding to isolates selected for whole genome sequencing are indicated by a black dot.

Table 1 summarizes the MLST alleles and sequence types (STs) associated with either the horse or intermediate group found among the 108 horse isolates analyzed. Forty-three STs were found among 108 horse isolates (Simpson’s index of diversity [SID] ± 95% confidence interval [CI], 0.962 ± 0.014) and the number of alleles at the individual loci ranged from 8 (for *mutS* and *atoB*) to 13 (*gtr*). Almost all STs and alleles of horse isolates were novel. Exceptions occurred with the alleles associated with ST10 and allele *mutS6*, which were previously detected in SDE causing human infections. The goeBURST analysis revealed 5 clonal complexes (CCs) at the single-locus-variant (SLV) level, while 15 STs were singletons (Fig. 1). Twenty STs were grouped into one CC (including 68 isolates), comprising isolates of both Lancefield groups, recovered in the four geographic locations and from all specimens (Supplementary Fig. S1). Analysis performed at triple-locus-variant (TLV) level showed that 36 STs (*n* = 100 isolates) were grouped into the major CC. Although Lancefield group C and group L isolates were associated with distinct STs, the latter exclusively found in STs 194 (*n* = 9 isolates), 210, 214 and 215 (*n* = 1 isolate each), all group L isolates had SLVs among group C isolates reflecting their shared alleles (Fig. 1).

**Figure 2:**
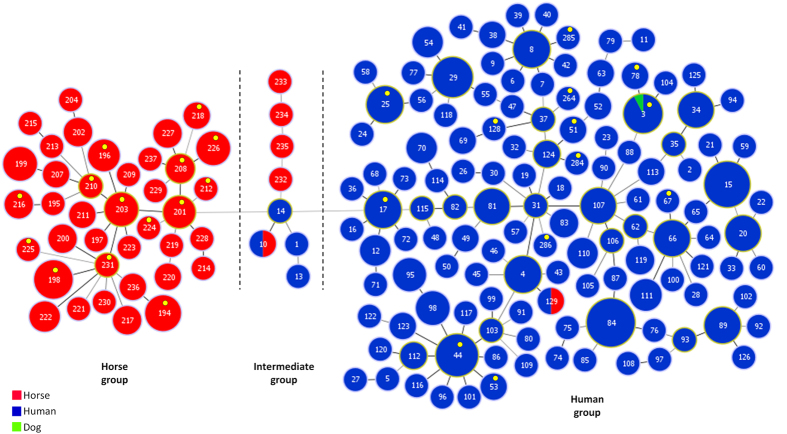
Minimum spanning tree representation of the relationships between 483 *S. dysgalactiae* isolates recovered from humans, horses and a dog. Dashed lines indicate the division in three main groups, corresponding to a difference of at least six different loci. The size of each circle is proportional to the number of isolates with that particular ST on a logarithmic scale. Putative CC founders are identified by a light green circle and putative sub-founders by a light brown circle (see legend of Fig. 1). STs corresponding to the whole genome sequences available are indicated by a yellow dot.

The goeBURST minimum spanning tree representing the relationships of STs derived from horse and all other STs showed that most STs clustered according to their host of origin (Fig. 2). Two main groups could be defined, one including 38 of the 43 horse STs (the horse group) and one larger group including almost exclusively human isolates (the human group). None of the 108 horse isolates from our collection was included in the human group, but two SDE isolates, one from a dog and another from a horse, reported previously^18^,^19^ were included in this group. No allele at any of the seven loci used for MLST was shared between members of the horse and human groups. A third smaller group of eight STs (here designated as the intermediate group), including isolates from humans (*n* = 4) and horses (*n* = 5), also had a high number of unique alleles at the seven loci, but shared the *atoB8* allele with the horse group, and *gtr4* and *recP12* alleles with the human group (Fig. 2).

Neighbor-joining trees built for each of the individual loci used in the MLST scheme showed that the alleles of the horse and intermediate group were frequently more closely related to each other than to the alleles found among human isolates, although bootstrapping failed to support this distinction (Supplementary Fig. S2). Only in the tree constructed from the seven concatenated loci were all the sequences of the horse and intermediate groups joined together in a single branch with 100% bootstrap support (Supplementary Fig. S2), while the human isolates were not clustered into a distinct monophyletic group.

### *emm* types of horse isolates

Nineteen distinct *emm* types were found among horse isolates (Table 2), seven of which were novel. An *emm* gene could be amplified by PCR from 60 (55.6%) isolates, while an *emm* type was assigned in additional five isolates which were *emm* non-typable by PCR but had their genome sequenced. Failure to amplify the *emm* gene in these isolates was possibly due to reduced sequence similarity with the pair of primers used for *emm* typing, since several mismatches in the primers’ annealing regions could be identified.

The most common *emm* type, *stC210,* was found in 16 isolates of six different MLST STs, and four other *emm* types were also associated with more than one ST. Isolates of a given ST usually showed a single *emm* type, although two distinct *emm* types were found among ST199 and ST216 isolates, and several STs included *emm* typable and non-typable isolates. *emm* types were neither shared between Lancefield group C and L isolates, nor between members of the horse and intermediate groups (*stC7505* and *stL2764* were the *emm* types exclusively associated to the latter). A single horse isolate had *emm* type *stG6*, the only *emm* type also found among isolates of the human group, while a second isolate had a new *emm* type, *stG14*, which had 75% sequence identity to *stG6* in the 150 bases encoding the first 50 residues of the mature M protein targeted by *emm* typing. No other sequence similarities were evident between horse and human *emm* types.

Whole-genome analysis showed that all 14 horse isolates had an *emm* gene in the same genomic location (Supplementary Fig. S3). In two isolates, a transposase followed by a second *emm*-like gene was present immediately downstream of the *emm* gene, but only the first gene was amplified by conventional *emm* typing. The gene arrangement around the *emm* locus was more similar to the one found in human SDE than SDD, including the presence of a streptokinase gene in all horse isolates which is absent from the SDD genome. Upstream of the *emm* locus the gene arrangement is also conserved among horse isolates and identical to human SDE in a region of nearly 12 Kb encompassing 11 genes. This similarity is interrupted by the presence of a gene encoding a putative internalin A-like precursor in horse isolates which is absent from SDD or human SDE isolates. BLAST revealed that the internalin A-like alleles present in equine *S. dysgalactiae* presented 93 to 96% DNA sequence and amino acid identity to a histidine triad protein found in *Streptococcus equi* subsp. *zooepidemicus* and *Streptococcus equi* subsp. *equi* genomes, with no other significantly similar sequences found in other genomes. The highest scoring hit was observed in the *S. equi* subsp. *zooepidemicus* MGCS10565 genome (GenBank accession number CP001129.1, 95% amino acid identity, Fig. S4), in which the internalin A-like gene is flanked by the same genes found in *S. dysgalactiae* horse isolates.

### Relationship to *S. dysgalactiae* isolated from human and animal infections

**Figure 3:**
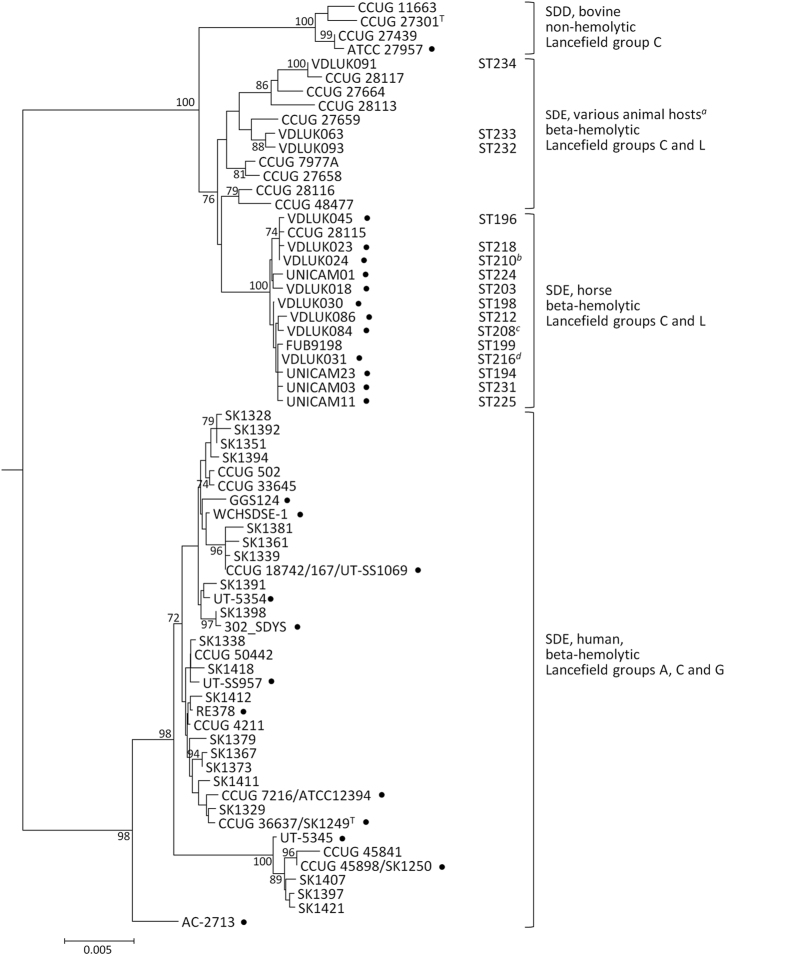
Neighbor-joining tree of *S. dysgalactiae* based on the concatenated sequences of the seven genes used in MLSA. Fifty-seven unique concatenated sequences (3063 bp each) were included in the analysis. The corresponding MLST STs are indicated for the 19 horse isolates newly analyzed in this study; ^*a*^includes also isolates from dogs (*n* = 5), pigs (*n* = 3) and horse (*n* = 1)^12^; ^*b*^concatenated sequence is identical to isolates VDLUK009 (ST226) and CCUG 28114^12^; ^*c*^concatenated sequence is identical to isolate CCUG 28112^12^; ^*d*^concatenated sequence is identical to isolate LMG15901 (ST200) and UNICAM18 (ST201); ^T^, denotes type strain. The sequence of the *S. canis* type strain CCUG 27661^12^ was used to root the tree.

We sought to clarify the phylogenetic position of the horse isolates within the *S. dysgalactiae* taxon by MLSA. The neighbor-joining tree of concatenated MLSA loci showed two distinct clades: one grouping human SDE isolates and the other all isolates of animal origin (Fig. 3). The four non-hemolytic SDD isolates included in the analysis acted as an outgroup within the animal clade, grouping together with a high bootstrap support. Although isolates from the MLST-based horse group were more closely related to SDD and beta-hemolytic *S. dysgalactiae* from other animals than to human SDE, they grouped into one tightly related branch with 100% bootstrap support. On the other hand, isolates with MLST STs of the intermediate group (STs 232 to 234) did not group within the horse or SDD branches, but were more closely related to beta-hemolytic isolates recovered from dogs (*n* = 5), pigs (*n* = 3) and horse (*n* = 1) previously described^12^. In contrast, the divergent branch present within the human clade and supported by a high bootstrap value could be explained by the presence of a highly disparate *rpoB* allele^12^. The segregation of alleles into three groups also occurred for the set of genes used for MLSA with horse isolates presenting unique alleles except for a single *pyk* allele (GenBank accession number JN632108) which was shared between two horse isolates and pig isolate CCUG28113.

Six 16S rRNA gene alleles were found among the 19 horse isolates which had their 16S rRNA gene sequenced. Five alleles were found exclusively associated with members of the horse group and among these only 4 nucleotide positions differed. These 16S rRNA gene alleles were more closely related to the 16S rRNA gene of the SDE type strain (GenBank accession number JN639432), from which these differed by only 1 to 3 bp across the 1262 bp analyzed, than to the SDD type strain (GenBank accession number JN639392) from which these differed by 14 to 16 bp. In contrast, the three isolates with MLST STs of the intermediate group presented a single 16S rRNA gene allele, which differed from SDD and SDE type strains by 6 and 11 bp, respectively. This allele was identical to the sequences derived from pig and dog isolates (GenBank accession numbers JN639382, JN639384 and JN639387) included in the analysis.

To clarify the taxonomic position of the horse isolates we performed an average nucleotide identity (ANI)^20^ analysis using the whole genome sequences available in GenBank. This revealed that while both horse and human isolates formed highly coherent groups (average ANI > 0.99, Supplementary Fig. S5) they differed more significantly between them with average ANI = 0.975, but with ANI values higher than those found when comparing either group with the type strain of SDD (average ANI ≈ 0.96, Supplementary Fig. S5). A preliminary analysis of the core genome of the horse and human isolates revealed that shared alleles occurred in only 5.1% (51/994) of the loci. This reinforces not only the different evolutionary history of these two populations, but also that if recombination between the core genomes occurs this is a rare event.

## Discussion

The taxonomic status of beta-hemolytic *S. dysgalactiae* isolated from horses and other animals has been controversial over the years since discrepant results have been obtained when assigning these strains to one of the two *S. dysgalactiae* sub-species^5^,^21^. While most 16S rRNA gene sequences determined for our horse isolates were in agreement with an SDE classification, MLSA indicated that all beta-hemolytic isolates of animal origin were more closely related to the SDD type strain than to human SDE, thus challenging their classification as SDE, as previously noted^12^. However, MLSA also indicates that strains currently classified as SDD are an outgroup within the animal clade (Fig. 3), in agreement with an old observation that the non-hemolytic strains of bovine origin were distinct from all other *S. dysgalactiae*^5^. Supporting this, the ANI values of the comparison of horse isolates with either human isolates or the type strain of SDD are well below those found when comparing isolates within each of the groups and close to the value obtained when comparing the two recognized subspecies. These differences between the archetypical SDD strains and other animal *S. dysgalactiae*, reinforced by the unique genotypic characteristics of our horse isolates, suggest that the archetypical strains of the two subspecies currently recognized may not accurately reflect the diversity of the *S. dysgalactiae* taxon. Future studies analyzing a greater number of bovine SDD and SDE from diverse animal hosts (other than horses) are needed, in order to clarify all the potential divisions within this species before a definite decision on how to classify these isolates can be made.

The characterization by MLST showed that horse and human isolates were clearly separated from each other, since equine isolates were associated with MLST alleles and STs that were, with very few exceptions, distinct from those previously published for human SDE. Members of the MLST/goeBURST-defined horse group (Fig. 2) showed complete allele distinction relative to human SDE in MLST, MLSA and 16S rRNA gene loci, strongly suggesting they represent isolated populations, with limited genetic exchange. MLSA was instrumental in showing that members of the horse group were also distinct from *S. dysgalactiae* strains recovered from other animal hosts, as they grouped into a unique, tightly related branch, within the animal clade (Fig. 3). Taken together, these results strongly suggest the existence of a unique population in horses, which is the first evidence of a host-specific genomovar among beta-hemolytic *S. dysgalactiae* from animals.

Our data show that horses are mainly (but not exclusively) affected by this specific variant (n = 103/108, 95%). goeBURST suggested a recent clonal origin of the horse genomovar, since most STs were linked at the SLV level and all but two STs of the horse group (STs 218 and 225) were related at TLV level. This was concordant with the monophyletic origin evidenced by the MLSA analysis (Fig. 3). Epidemiologically unrelated isolates from the US and three European countries were included in the major CC, suggesting that a low genetic diversity can be a general feature of this population. Thus, it is likely that the horse isolates represent variants of a clone recently adapted to the equine host, similarly to *S. equi* subsp. *equi,* another Lancefield group C streptococci which is a strict horse pathogen believed to have evolved from an *S. equi* subsp. *zooepidemicus* ancestor^22^. While *S. equi* subsp. *equi* infects mainly the equine upper respiratory tract, the *S. dysgalactiae* genomovar causes distinct clinical conditions in this host^7^,^10^ and the current analysis did not unveil any specific tissue tropism for the different STs (Supplementary Fig. S1), though a predominance of reproductive tract infections was noted.

The existence of a second *S. dysgalactiae* group causing equine infections was suggested by the intermediate group of STs associated with some isolates recovered from horses and human invasive infections in the United States^15^. MLSA and 16S rRNA gene analysis showed that these isolates were more similar to beta-hemolytic isolates from dogs, pigs and horses reported elsewhere^12^, raising the possibility that a set of genetically related strains co-exist in humans, horses and other animal hosts. Although SDE infection in humans is generally thought to have an endogenous origin^4^,^11^, two recent studies reported SDE transmission between humans and animals. In Australia, an identical SDE strain was found being carried by a child and a dog in an Aboriginal community^18^, while a study from Brazil made a similar finding involving horses^19^. Interestingly, in both studies the isolates exhibited genotypic features typical of the human SDE population, as shown by their inclusion in the MLST-defined human group (ST3 and ST129, Fig. 2) suggesting cross-species transmission in the human to animal direction. This was not the case with ST10, the other ST shared by horse and human isolates. The preferential hosts of isolates belonging to the intermediate group, which includes ST10, is unclear at the moment, but the genotypic differentiation from most human SDE raises the possibility of a zoonotic origin of these isolates. The study of a greater number of isolates of animal origin is needed, as human infection by this group of strains may pass essentially unrecognized unless there is a detailed genotypic characterization of this group.

The extensive use of *emm* typing for characterizing SDE human isolates contrasts with its limited application in *S. dysgalactiae* from animal sources, given that the latter are often *emm* non-typeable^12^,^23^. Accordingly, nearly half of the horse isolates could not be typed by the *emm* typing protocol established for GAS and human SDE, even though the *emm* locus seems to be conserved in these isolates as suggested by whole-genome analysis. The segregation of the horse population was also evident by its association with novel *emm* types or with *emm* types previously identified among horse isolates (ftp://ftp.cdc.gov/pub/infectious_diseases/biotech/tsemm/). The notable exception occurred with a group L isolate (ST215) which had *emm* type *stG6*, one of the most common *emm* types among human SDE^2^,^3^,^14^. Since all seven MLST alleles of this isolate were typical of the horse variant, it is likely it has acquired this specific *emm* type by horizontal gene transfer. This might also be the case with an *stG6*-related (*stG14*) *emm* type present in another isolate. A third *emm* type (*emm229*), found in four ST199 isolates, is an infrequent *emm* type of GAS found in the Northern Territory of Australia (http://spyogenes.mlst.net/ and http://www.cdc.gov/streplab/M-ProteinGene-typing.html), raising the possibility that *emm* genes typical of SDE may also be acquired by GAS.

The gene arrangement around the *emm* locus in horse isolates resembled that found in human SDE, suggesting that the M-like protein or the streptokinase encoded at this locus, may contribute to virulence in the equine host, in line with their proposed roles in human infections^4^. The horse tropism shown by this *S. dysgalactiae* genomovar may be driven not only by the presence of specific virulence factors but also by the unique properties of virulence factors they have in common with other SDE. For instance, it was been shown that streptokinases secreted by human and horse isolates demonstrate species-specific activation of plasminogen^24^. On the other hand, the similarity of the internalin A-like gene present in the horse genomovar with the genes found in the two other major streptococcal horse pathogens^7^,^10^ is highly suggestive of an important role of this protein in pathogenesis, although this remains to be demonstrated.

In conclusion, our study shows that *S. dysgalactiae* isolated from horses represent a previously unrecognized horse-specific genomovar. We describe the unique genotypic properties associated with this variant, including MLST STs, allele sequences at distinct MLST *loci* and *emm* types (indicated in Tables 1 and 2) allowing their identification in future studies. This genomovar can also be identified by its specific MLSA loci and 16S rRNA gene alleles, which showed minimal variation within the set analyzed. Further studies will be instrumental in expanding the known genetic diversity of this horse population, in identifying potential factors responsible for the tropism for this particular host and in clarifying the frequency of transmission of these strains to other hosts.

## Methods

### Bacterial isolates

A total of 108 *S. dysgalactiae* isolates from horses were studied. Eighty-seven isolates were recovered at the University of Kentucky, United States, from 1981 to 2011, 18 isolates were recovered at the University of Camerino, Italy, from 2006 to 2009, and 1 isolate was recovered at the Freie Universität Berlin, Germany, in 2004. Two isolates, LMG15833 (CCUG 28114) and LMG15901 (CCUG 28118), originally recovered in Sweden in 1989, were obtained from the BCCM/LMG collection (Gent, Belgium). Most of the isolates were associated with infections of the reproductive tract, being recovered from uterine (*n* = 42), fetal/placenta (*n* = 26) and vaginal specimens (*n* = 3). Other sources included the respiratory tract (*n* = 16, including isolates recovered from animals that died from other causes), skin and soft tissue (*n* = 14) and deep tissues (*n* = 7, including liver [n = 2], bone [n = 2], brain, abdominal fluid and guttural pouch). Detailed description of the source of the isolates is given in Supplementary Table S1.

### Hemolysis and Lancefield grouping

Beta-hemolysis and colony size were confirmed in tryptic soy agar (Oxoid, Hampshire, England) supplemented with 5% (vol/vol) defibrinated sheep blood, after overnight incubation at 37 °C. The Lancefield group was determined by a commercial latex agglutination technique (Streptococcal Grouping Kit, Oxoid, Basingstoke, United Kingdom). The Lancefield group L was confirmed by using a different rabbit serum coupled to latex particles (Statens Serum Institut, Copenhagen, Denmark).

### MLST analysis

All isolates were characterized using the MLST scheme available for SDE (http://pubmlst.org/sdysgalactiae/), which uses internal fragments of the seven housekeeping genes *gki*, *gtr*, *murI*, *mutS*, *recP*, *xpt* and *atoB* (also called *yqiZ*)^17^. Unique sequences at each locus were assigned allele numbers. The combination of the seven allele numbers for each isolate was used to define STs. The goeBURST algorithm implemented in PHYLOViZ^25^ was used to establish relationships between STs. CCs were defined from SLV up to TLV level. Comparison between horse and human isolates was achieved by incorporating in the analysis the MLST data previously published for 359 SDE isolates recovered from human specimens^15^,^16^,^17^, plus the MLST data extracted from 13 SDE genomes (http://www.ncbi.nlm.nih.gov/genome/823). Four additional SDE isolates recently found infecting simultaneously humans and either a dog^18^ or a horse^19^, were also included in the analysis. An expansion of the goeBURST algorithm was used to generate a minimum spanning tree reflecting possible relationships between horse and human derived MLST STs^25^. MEGA (version 5)^26^ was used to construct trees of the individual and concatenated MLST loci, by using the neighbor-joining and the Kimura two-parameter substitution model. Branch support was tested by 1,000 replicate bootstrap tests in each analysis. MLST sequence data from GAS and *S. canis* (which have equivalent MLST schemes) were used for comparison and were obtained from http://spyogenes.mlst.net/ and http://pubmlst.org/scanis/, respectively.

### Statistical analysis

The diversity of MLST classifications was quantitatively evaluated by calculating the SID with 95% CIs^27^ using the Comparing Partitions website (http://www.comparingpartitions.info).

### *emm* typing

Characterization of the 108 horse isolates by *emm* typing was carried out with the primers and conditions available at http://www.cdc.gov/streplab/protocol-emm-type.html, as previously described^14^.

### MLSA and 16S rRNA gene sequencing

In order to allow the comparison of horse isolates with *S. dysgalactiae* recovered from other animal hosts, a subset of 19 horse isolates with distinct STs (as determined by the MLST scheme described above) were chosen for analysis by MLSA^12^ and 16S rRNA gene sequencing^14^. Sequences of internal fragments of the housekeeping genes *map*, *pfl*, *ppaC*, *pyk*, *rpoB*, *sodA* and *tuf* used in MLSA were concatenated to originate a 3063 bp sequence for each isolate, while the 16S rRNA gene sequences were trimmed to 1262 bp. A neighbor-joining tree of the MLSA sequence was constructed as indicated for MLST. Both MLSA and 16S rRNA data obtained for this set of horse isolates was compared with the data from the study by Jensen *et al.*^12^, which analyzed 76 *S. dysgalactiae* isolates (61 human SDE and 15 isolates from distinct animal hosts, including 3 non-hemolytic SDD), and with the data from *S. dysgalactiae* genomes.

### Whole genome sequencing and ANI analysis

The genomes of 14 horse isolates of distinct STs (Figs 1 and 2) were sequenced using Illumina HiSeq. Whole genome comparisons were carried out using the genomes of 13 SDE human isolates and SDD ATCC 27957 available in GenBank (http://www.ncbi.nlm.nih.gov/genome/genomes/823). ANIm values^20^ were calculated for each pairwise comparison.

### Nucleotide sequence accession numbers

The sequences of the novel MLST alleles were submitted to GenBank under accession numbers KM652555 to KM652615 and to the SDE MLST database (http://pubmlst.org/sdysgalactiae/). Novel MLSA and 16S rRNA gene sequences were submitted to GenBank under accession numbers KM589383 to KM589401. The sequence of the novel *emm* types were submitted to the CDC database (http://www.cdc.gov/streplab/M-ProteinGene-typing.html). Whole genome sequences of *S. dysgalactiae* horse isolates were submitted to NCBI under BioProject number PRJNA321465.

## Additional Information

**How to cite this article**: Pinho, M. D. *et al.* Beta-hemolytic *Streptococcus dysgalactiae* strains isolated from horses are a genetically distinct population within the *Streptococcus dysgalactiae* taxon. *Sci. Rep.* **6**, 31736; doi: 10.1038/srep31736 (2016).

## Supplementary Material

Supplementary Information

## Figures and Tables

**Table 1:** MLST data of 108 *S. dysgalactiae* horse isolates.

ST/locus	Horse group		Intermediate group^a^		Total	
	Alleles	No. of partitions	Alleles	No. of partitions	No. of partitions	SID (±95% CI)
ST	194–204, 207–231^b^	38	10, 232–235^b^	5	43	0.962 (0.014)
*gki*	22–29	8	10, 30–32	4	12	0.452 (0.114)
*gtr*	23–32	10	4^c^, 33, 34	3	13	0.726 (0.061)
*murI*	20–26	7	7, 27, 28	3	10	0.400 (0.116)
*mutS*	23–25	3	6, 7, 26–28	5	8	0.423 (0.097)
*recP*	33–38	6	12^d^, 39, 40	3	9	0.273 (0.113)
*xpt*	41–43, 45–47, 49	7	13, 48	2	9	0.625 (0.064)
*atoB*	8, 24–28	6	8^e^, 29, 30	3	8	0.665 (0.054)

^a^STs 1, 13 and 14, and alleles *gtr5*, *gtr6*, *murI6* and *atoB9* were also exclusively associated with the intermediate group, but are absent from the table as they were not found among horse isolates.

^b^The STs resulting from the unique combinations of alleles at the seven loci are indicated.

^c^The *gtr4* allele was found in 2 STs of the intermediate group and 10 STs of the human group.

^d^The *recP12* allele was found in 5 STs of the intermediate group and in ST5 from the human group.

^e^The *atoB8* allele was found in 13 STs of the horse group and 4 STs of the intermediate group.

**Table 2:** *emm* types distribution among Lancefield groups and MLST STs of 108 *S. dysgalactiae* horse isolates.

*emm* type^a^	Lancefield group		STs (No. of isolates)	Total
	C	L		
*stC210*	16		226 (7), 196 (5), other^b^	16
*stC12*	10		202 (3), 208 (2), other^c^	10
*stG5063*	6		203 (6)	6
*stC1*		5	194 (5)	5
*emm229*	4		199 (4)	4
*stC37*	4		198 (4)	4
*stC14*	3		220 (2), 224 (1)	3
*stC13*	2		216 (2)	2
*stC8*	2		227 (2)	2
*stG15*	2		211 (2)	2
*stC11*	2		201 (1)^d^, 228 (1)	2
*stC16*	2		218 (1)^d^, 225 (1)^d^	2
*stC17*	1		212 (1)^d^	1
*stC7505*	1		10 (1)	1
*stG14*	1		216 (1)	1
*stG2574*	1		231 (1)	1
*stG6*		1	215 (1)	1
*stL1*		1	210 (1)^d^	1
*stL2764*	1		232 (1)	1
NT	38	5	198 (9), 201 (4), 222 (4), 194 (4), 217 (3), other^e^	43
Total	96	12	na^f^	108

^a^*emm* types are given by decreasing order of frequency; NT, non-typeable, which included isolates for which no PCR product could be obtained (*n* = 29) or unspecific amplification of more than one PCR product occurred (*n* = 14).

^b^included 4 STs with one isolate (STs 197, 213, 221 and 229).

^c^included 5 STs with one isolate (STs 199, 200, 204, 230 and 237).

^d^*emm* type assignement by whole genome sequence, since the isolate was non-typeable by PCR.

^e^included 4 STs with two isolates (STs 199, 200, 207 and 236) and 11 STs with one isolate (STs 195, 208, 209, 214, 218, 219, 223, 227, 233, 234 and 235).

^f^na, not applicable.
