# Supplementary material for: Beta-hemolytic Streptococcus dysgalactiae strains isolated from horses are a genetically distinct population within the Streptococcus dysgalactiae taxon
Source: Sci Rep. 2016 Aug 17;6:31736. doi: 10.1038/srep31736 (PMC4987641; doi:10.1038/srep31736)
Supplement: Supplementary Information [file srep31736-s1.pdf]

Beta-hemolytic Streptococcus dysgalactiae strains isolated from horses are a genetically distinct population within the Streptococcus dysgalactiae taxon

Marcos D. Pinho[1†], Erdal Erol[2†], Bruno Ribeiro-Gonçalves[1], Catarina I. Mendes[1], João A. Carriço[1], Sandra C. Matos[1], Silvia Preziuso[3], Antina Luebke-Becker[4], Lothar H. Wieler[4,5], Jose Melo-Cristino[1] and Mario Ramirez[1*]

[1] Instituto de Microbiologia, Instituto de Medicina Molecular, Faculdade de Medicina, Universidade de Lisboa, Lisbon, Portugal.

[2] Department of Veterinary Science, Veterinary Diagnostic Laboratory, University of Kentucky, Lexington, Kentucky, United States.

[3] Department of Veterinary Medical Sciences, University of Camerino, Matelica, Italy.

[4] Institute of Microbiology and Epizootics, Freie Universität Berlin, Germany.

[5] Robert Koch-Institute, Berlin, Germany.

†These authors contributed equally to this work
*Corresponding author:
Mario Ramirez
Instituto de Microbiologia
Faculdade Medicina
Universidade de Lisboa
Av. Prof. Egas Moniz
PT 1649-028 Lisboa
Portugal
Email: ramirez@fm.ul.pt
Tel: +351- 21 799 9460
Fax: +351- 21 799 9459

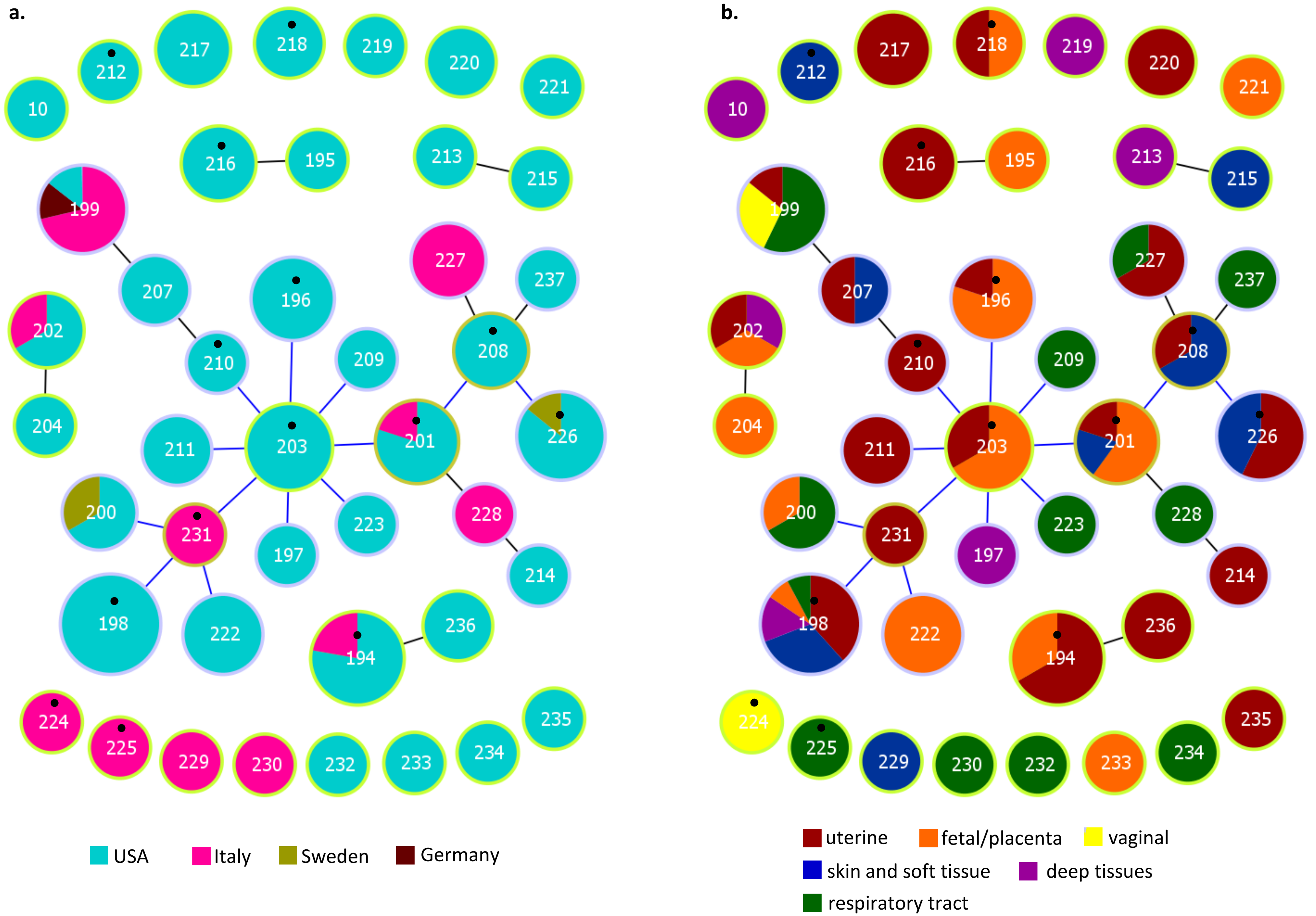

Figure S1. goeBURST diagram of 108 *S. dysgalactiae* horse isolates depicting the geographic location and the source of the isolates
The size of each circle is proportional to the number of isolates with that particular ST on a logarithmic scale. The number of isolates with the same characteristic is proportional to the respective color. STs assigned to the same CC at the SLV level are linked by straight lines. Putative CC founders are identified by a light green line and putative sub-founders by a light brown line. STs corresponding to isolates selected for whole genome sequencing are indicated by a black dot.

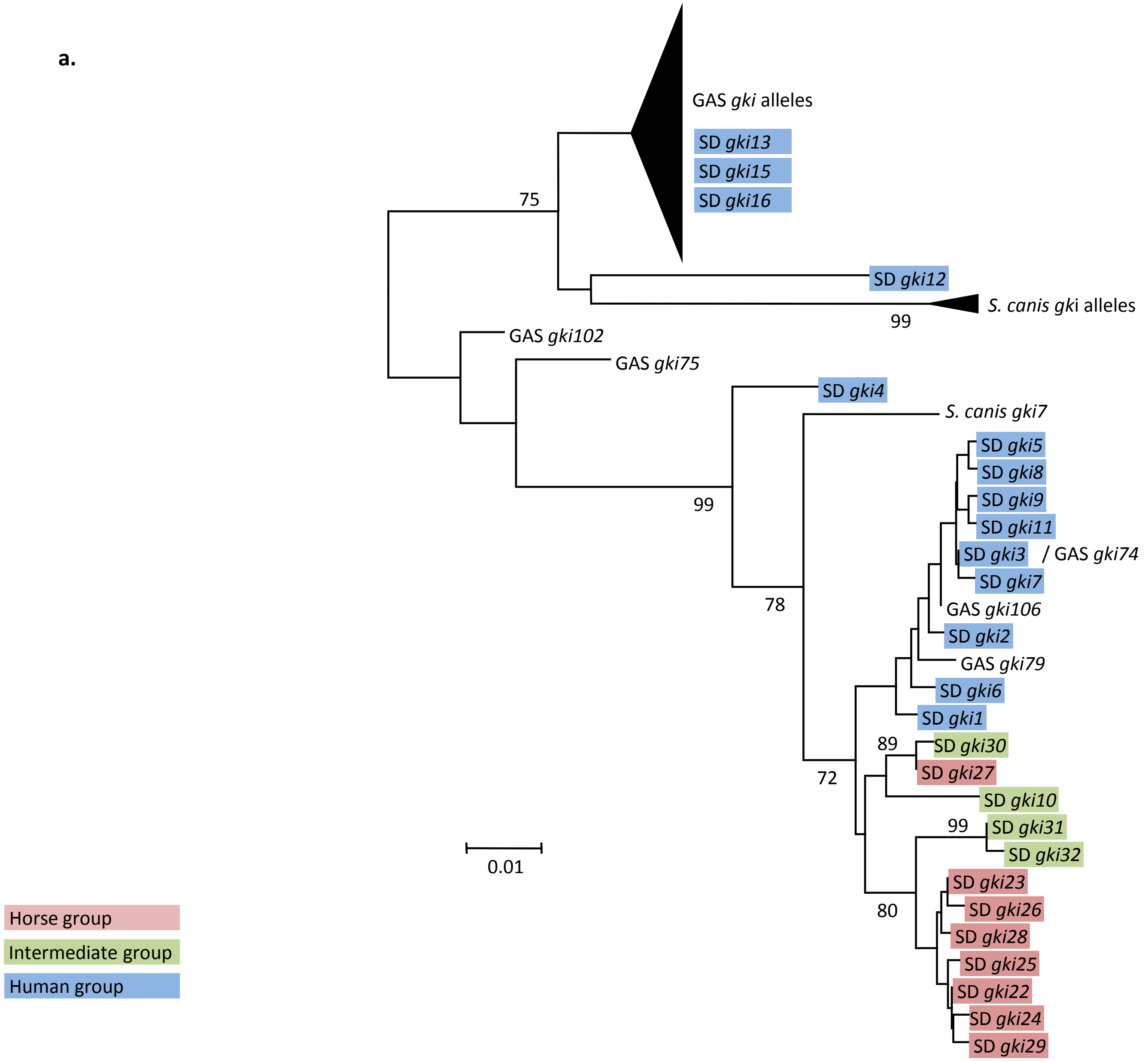

Figure S2. Neighbor-joining trees of *gki* (a), *gtr* (b), *murI* (c), *mutS* (d), *recP* (e), *xpt* (f), *atoB* (g) and six-gene concatenated alleles (2700 bp) (h) of *Streptococcus dysgalactiae* (SD), *S. canis* and GAS. The trees were built with the methods indicated in materials and methods. The association of SD alleles and STs with the three MLST defined groups are indicated by different colors.

b.

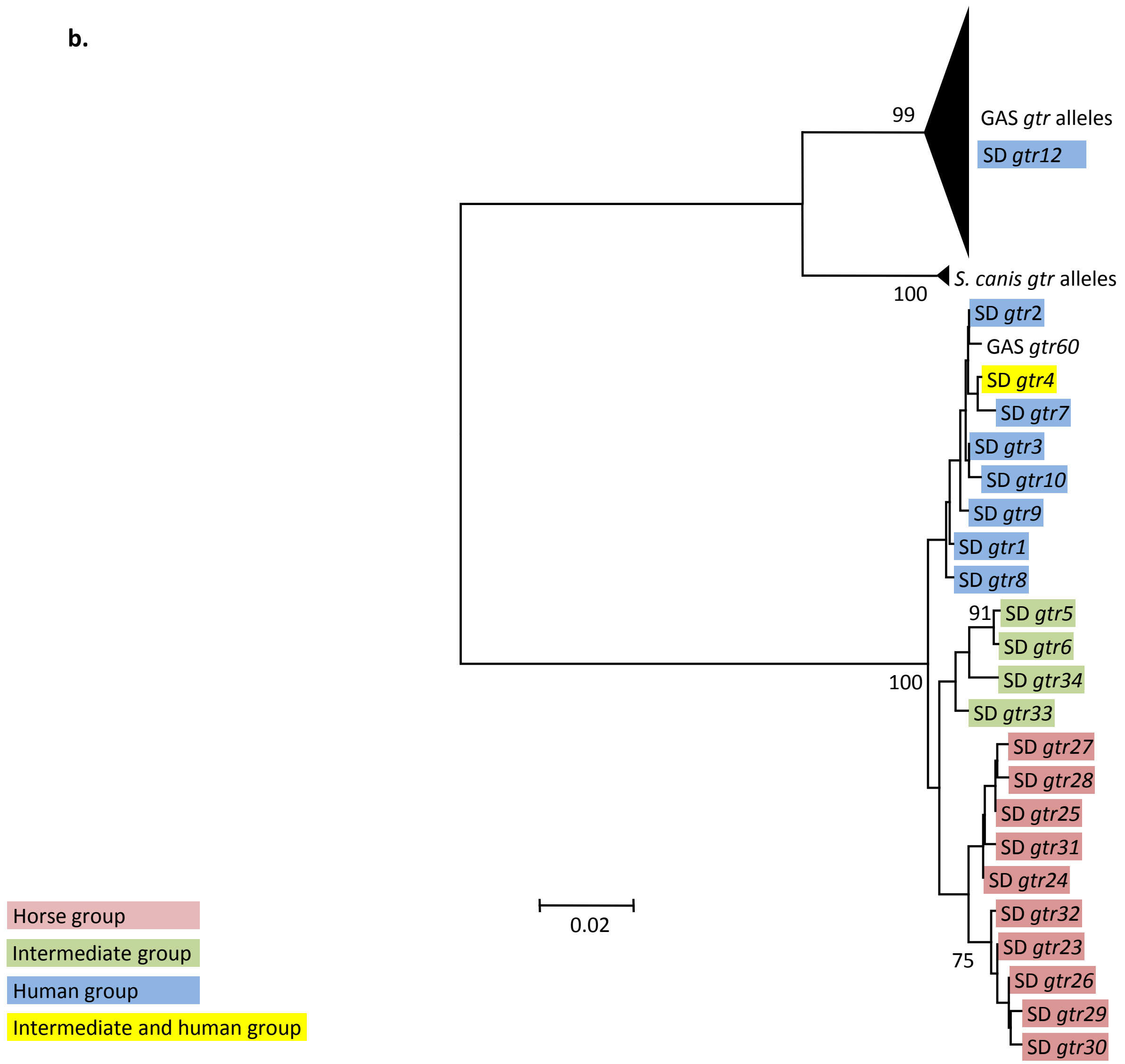
99
GAS gtr alleles
SD gtr12
S. canis gtr alleles
100
SD gtr2
GAS gtr60
SD gtr4
SD gtr7
SD gtr3
SD gtr10
SD gtr9
SD gtr1
SD gtr8
91
SD gtr5
SD gtr6
SD gtr34
100
SD gtr33
SD gtr27
SD gtr28
SD gtr25
SD gtr31
SD gtr24
SD gtr32
SD gtr23
75
SD gtr26
SD gtr29
SD gtr30
0.02
Horse group
Intermediate group
Human group
Intermediate and human group

c.

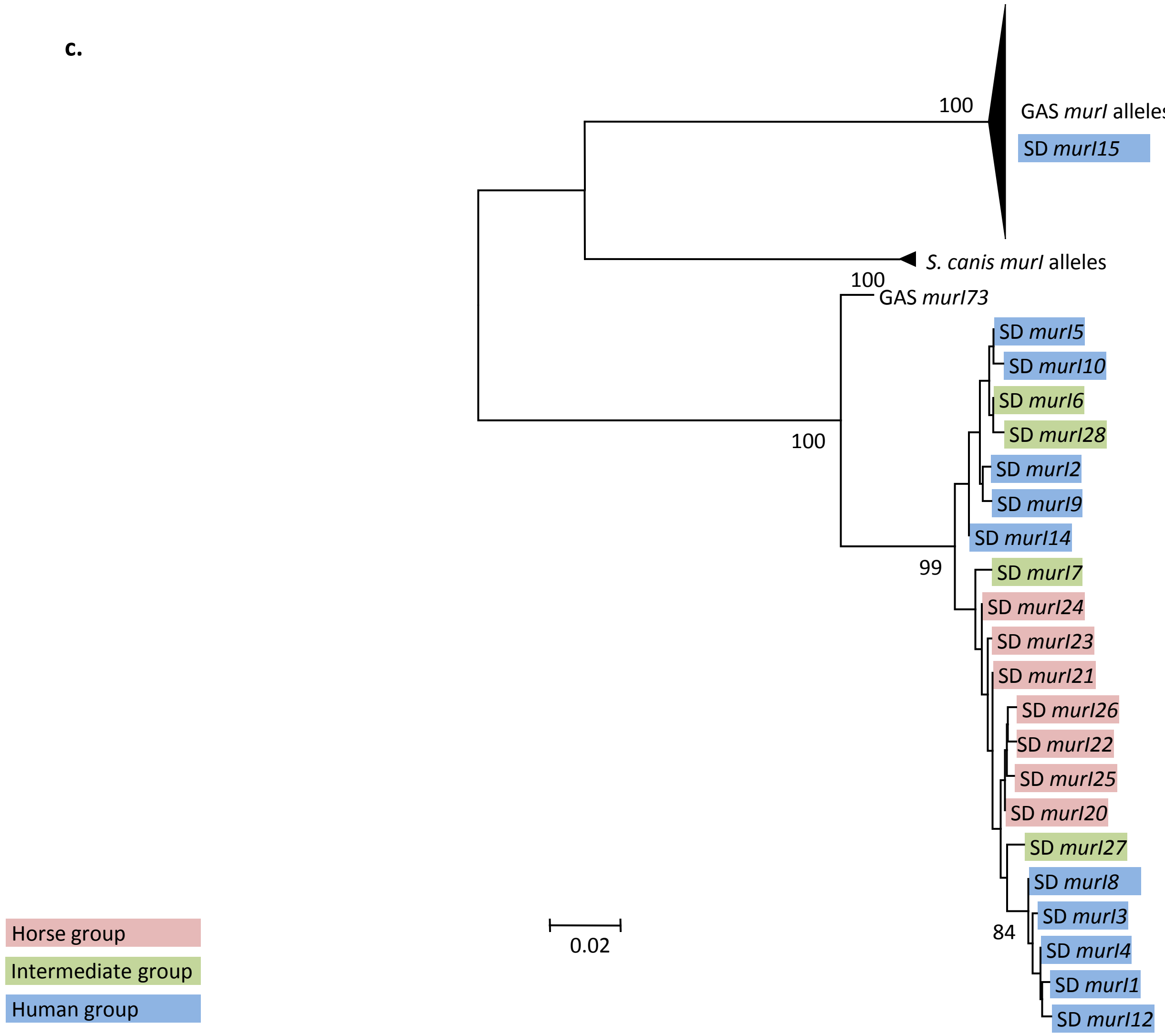
100
GAS *murI* alleles
SD *murI15*
S. canis murI alleles
100
GAS *murI73*
SD *murI5*
SD *murI10*
SD *murI6*
SD *murI28*
SD *murI2*
SD *murI9*
SD *murI14*
100
99
SD *murI7*
SD *murI24*
SD *murI23*
SD *murI21*
SD *murI26*
SD *murI22*
SD *murI25*
SD *murI20*
SD *murI27*
SD *murI8*
SD *murI3*
84
SD *murI4*
SD *murI1*
SD *murI12*
0.02
Horse group
Intermediate group
Human group

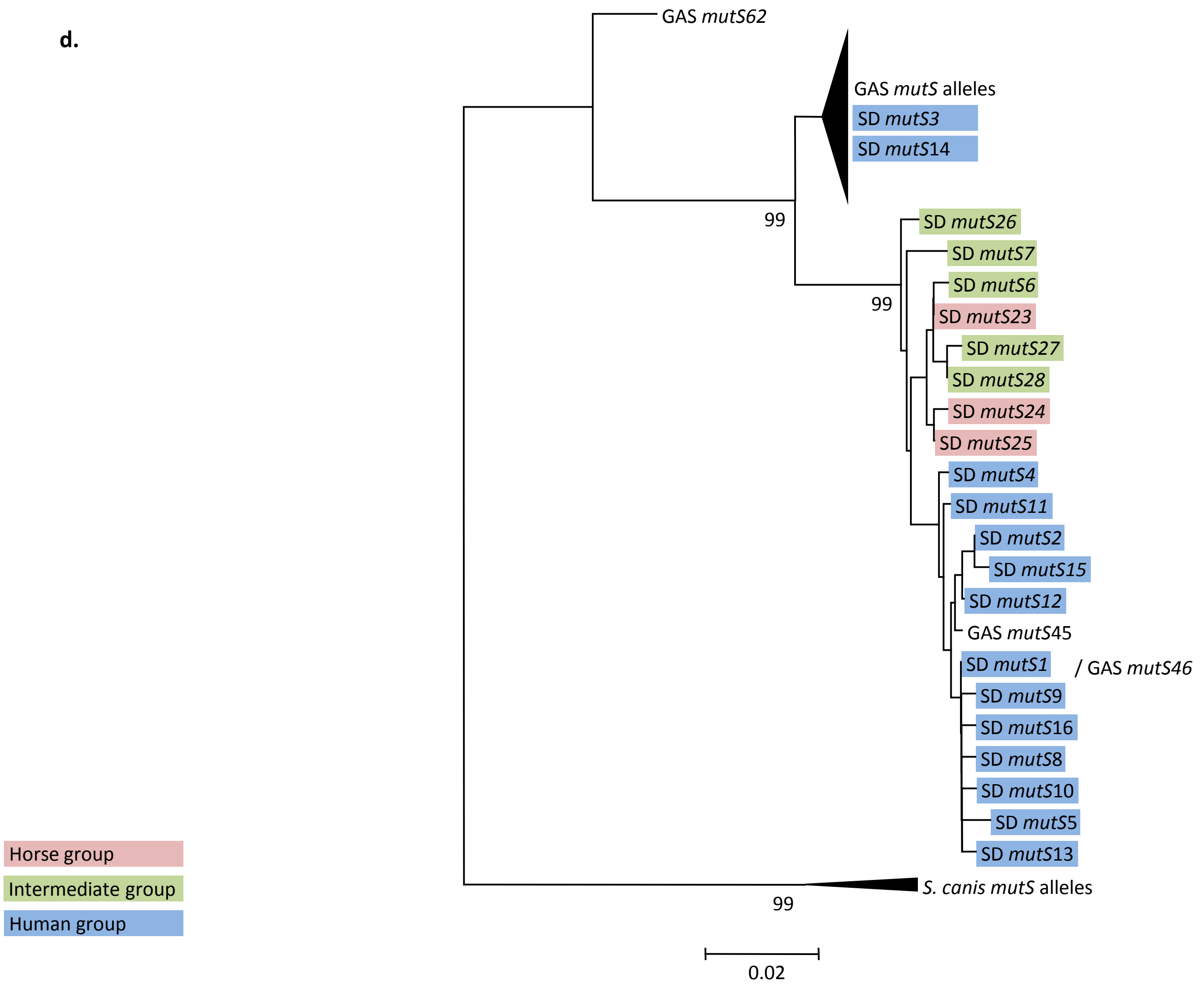
d.
GAS mutS62
GAS mutS alleles
SD mutS3
SD mutS14
99
SD mutS26
SD mutS7
SD mutS6
SD mutS23
SD mutS27
SD mutS28
SD mutS24
SD mutS25
99
SD mutS4
SD mutS11
SD mutS2
SD mutS15
SD mutS12
GAS mutS45
SD mutS1
/ GAS mutS46
SD mutS9
SD mutS16
SD mutS8
SD mutS10
SD mutS5
SD mutS13
S. canis mutS alleles
99
0.02
Horse group
Intermediate group
Human group

e.

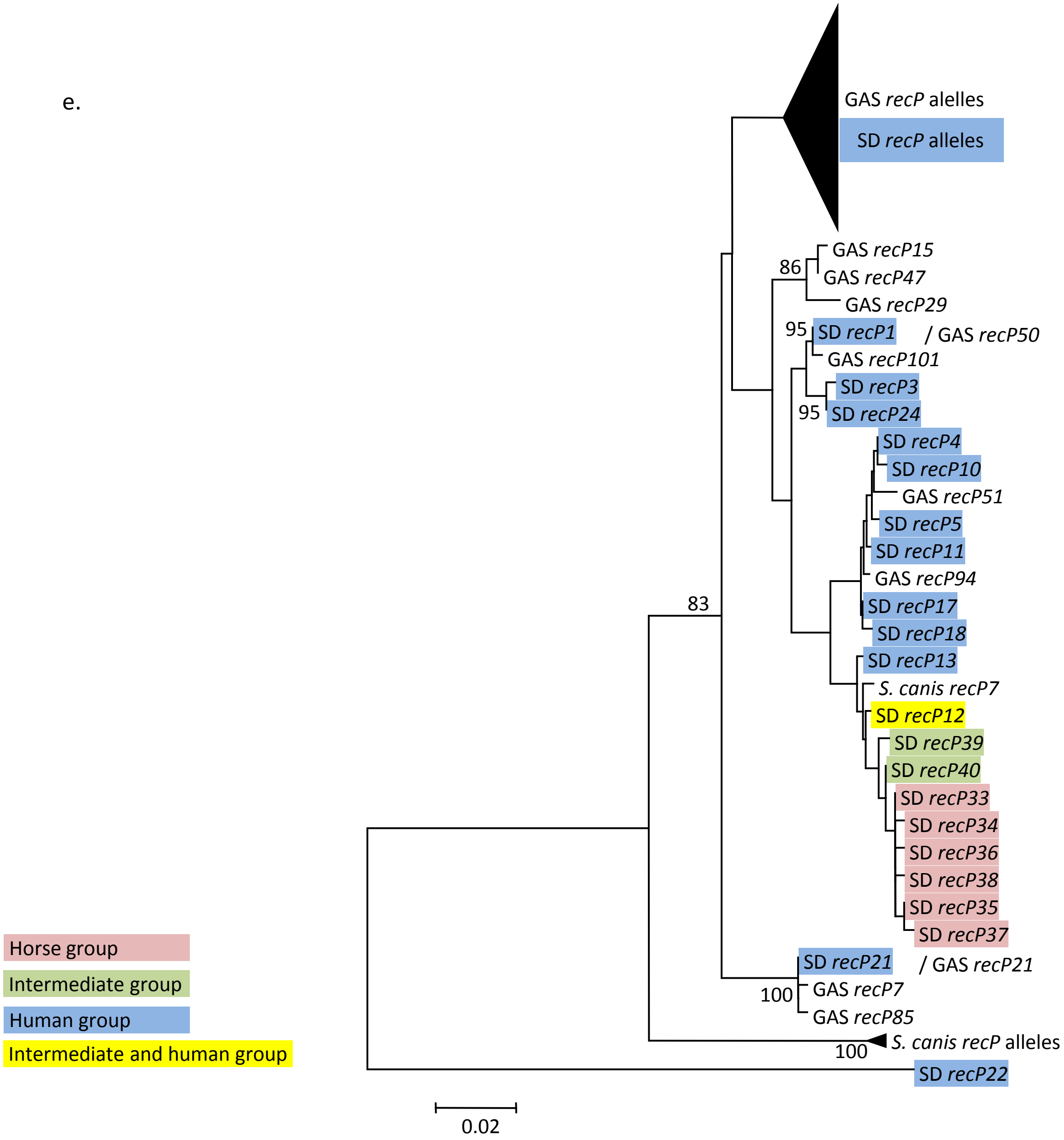
GAS recP alelles
SD recP alleles
86
GAS recP15
GAS recP47
GAS recP29
95
SD recP1 / GAS recP50
GAS recP101
SD recP3
95
SD recP24
SD recP4
SD recP10
GAS recP51
SD recP5
SD recP11
GAS recP94
SD recP17
SD recP18
SD recP13
S. canis recP7
SD recP12
SD recP39
SD recP40
SD recP33
SD recP34
SD recP36
SD recP38
SD recP35
SD recP37
83
SD recP21 / GAS recP21
100
GAS recP7
GAS recP85
100
S. canis recP alleles
SD recP22
0.02
Horse group
Intermediate group
Human group
Intermediate and human group

f.

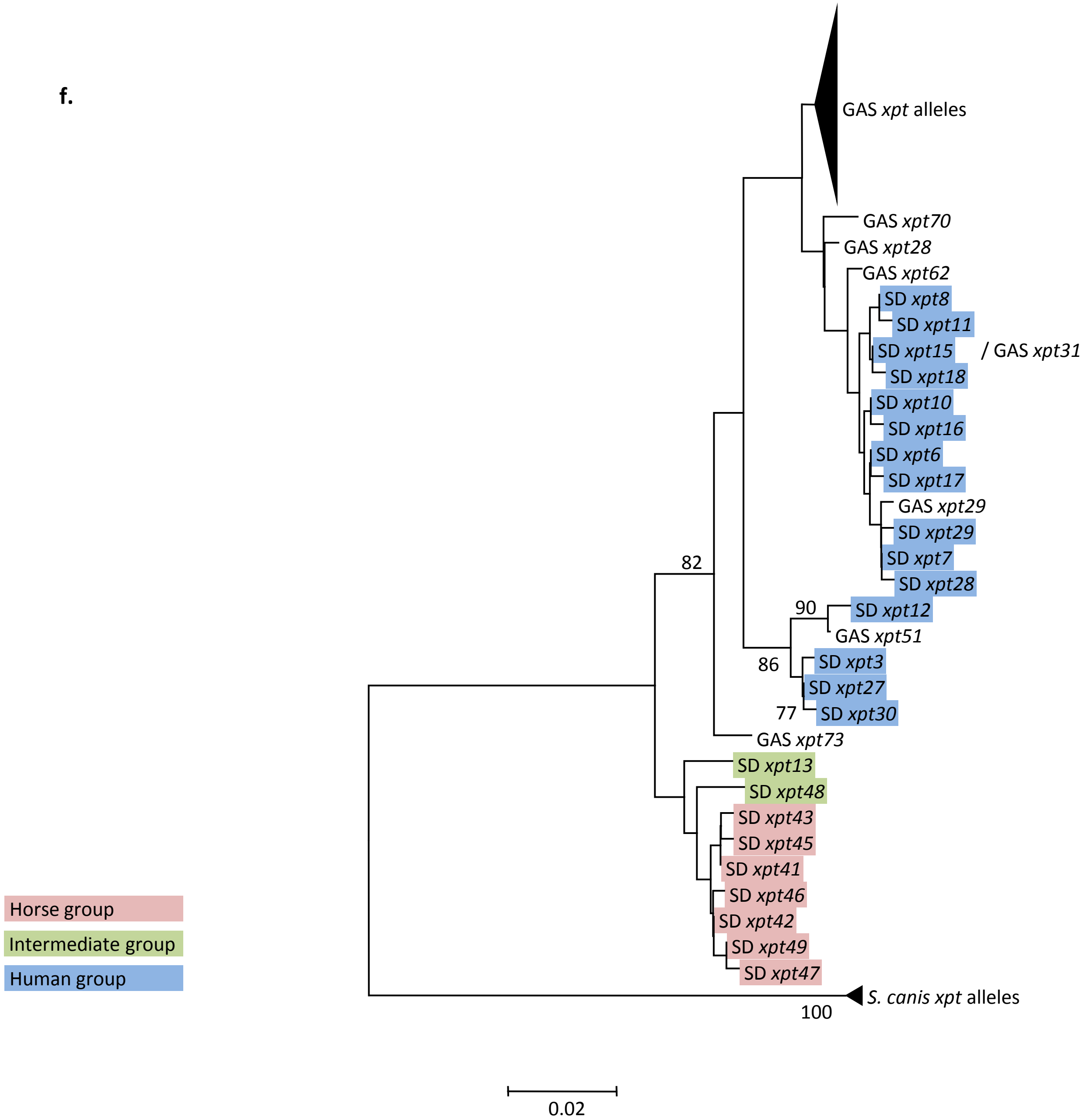

GAS xpt alleles
GAS xpt70
GAS xpt28
GAS xpt62
SD xpt8
SD xpt11
SD xpt15
/ GAS xpt31
SD xpt18
SD xpt10
SD xpt16
SD xpt6
SD xpt17
GAS xpt29
SD xpt29
SD xpt7
SD xpt28
82
90
SD xpt12
GAS xpt51
86
SD xpt3
SD xpt27
77
SD xpt30
GAS xpt73
SD xpt13
SD xpt48
SD xpt43
SD xpt45
SD xpt41
SD xpt46
SD xpt42
SD xpt49
SD xpt47
S. canis xpt alleles
100
0.02
Horse group
Intermediate group
Human group

g.

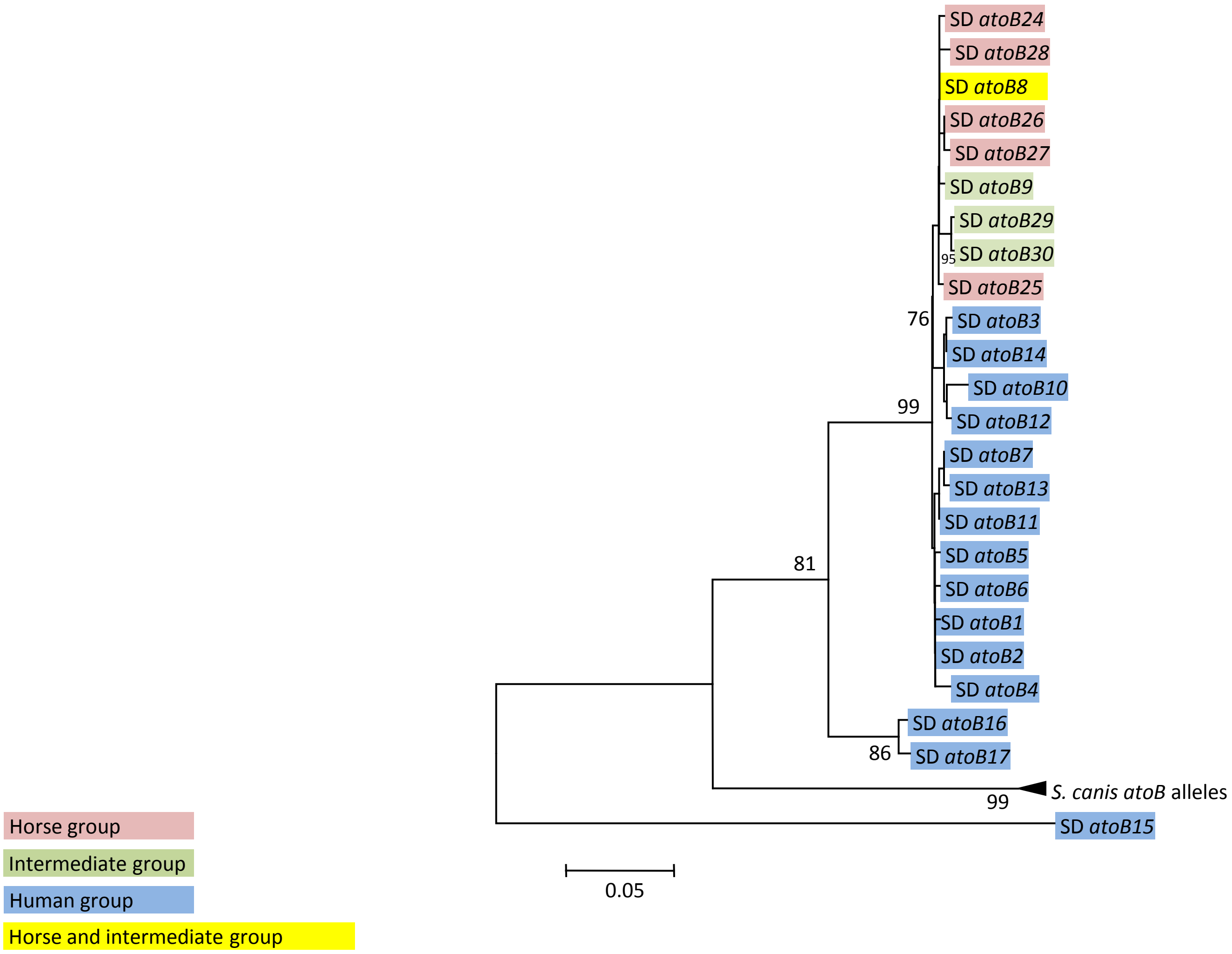
SD atoB24
SD atoB28
SD atoB8
SD atoB26
SD atoB27
SD atoB9
SD atoB29
95
SD atoB30
SD atoB25
76
SD atoB3
SD atoB14
SD atoB10
99
SD atoB12
SD atoB7
SD atoB13
SD atoB11
81
SD atoB5
SD atoB6
SD atoB1
SD atoB2
SD atoB4
SD atoB16
86
SD atoB17
S. canis atoB alleles
99
SD atoB15
0.05
Horse group
Intermediate group
Human group
Horse and intermediate group

h.

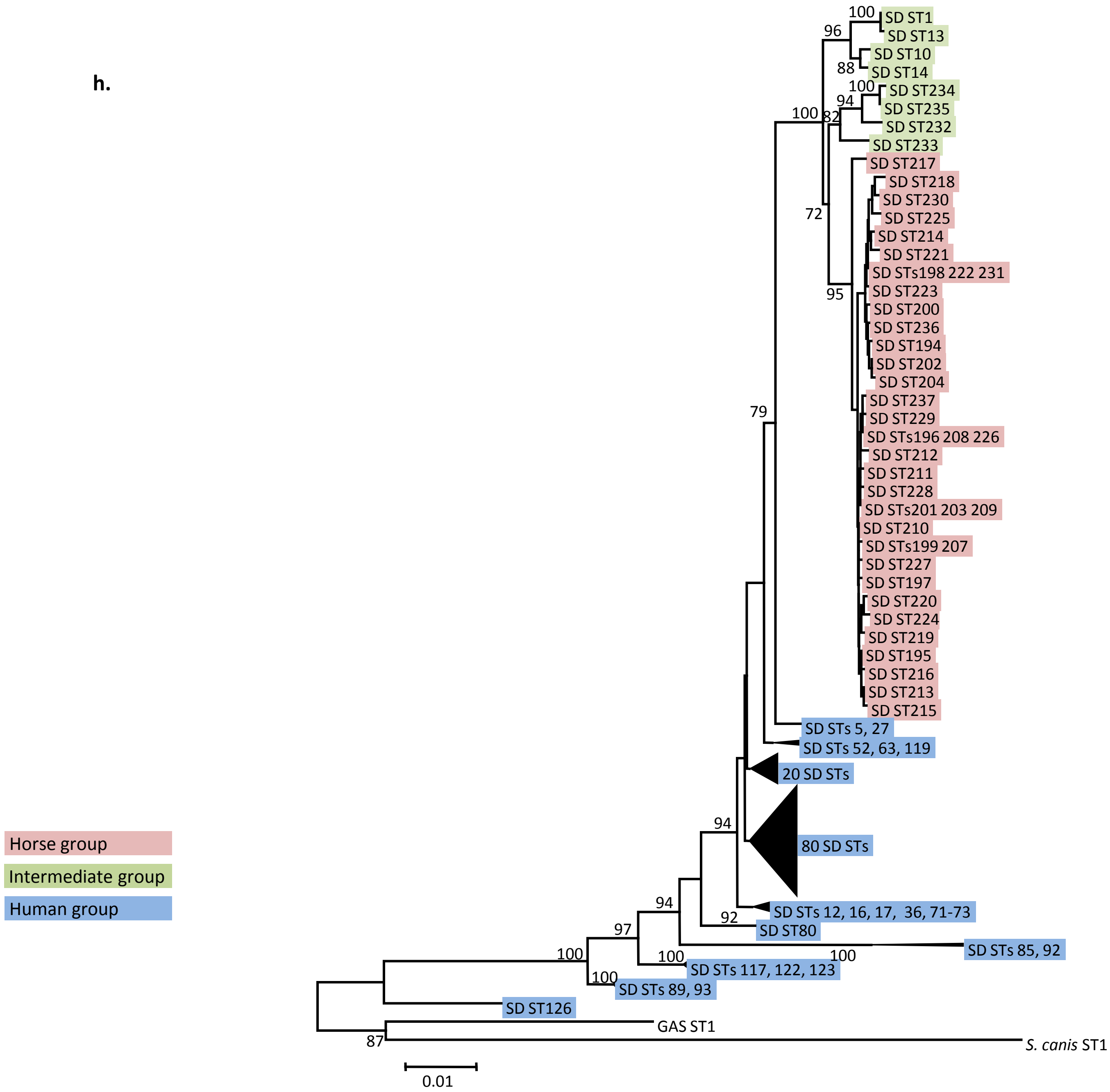
SD ST1
SD ST13
SD ST10
SD ST14
SD ST234
SD ST235
SD ST232
SD ST233
SD ST217
SD ST218
SD ST230
SD ST225
SD ST214
SD ST221
SD STs198 222 231
SD ST223
SD ST200
SD ST236
SD ST194
SD ST202
SD ST204
SD ST237
SD ST229
SD STs196 208 226
SD ST212
SD ST211
SD ST228
SD STs201 203 209
SD ST210
SD STs199 207
SD ST227
SD ST197
SD ST220
SD ST224
SD ST219
SD ST195
SD ST216
SD ST213
SD ST215
SD STs 5, 27
SD STs 52, 63, 119
20 SD STs
80 SD STs
SD STs 12, 16, 17, 36, 71-73
SD ST80
SD STs 85, 92
SD STs 117, 122, 123
SD STs 89, 93
SD ST126
GAS ST1
S. canis ST1
0.01
Horse group
Intermediate group
Human group

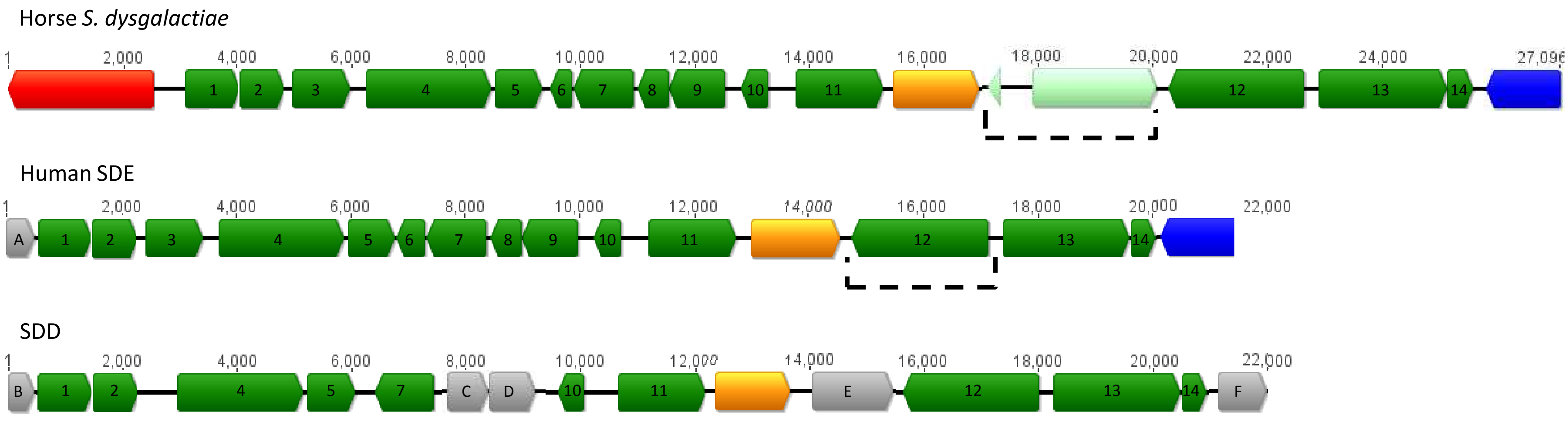

- internalin A-like gene
- M protein gene
- streptokinase gene
- transposase and M protein gene
- genes common to distinct groups

1. ribosomal protein L11 methyl transferase [NC_018712.1]
2. hypothetical protein [NC_018712.1]
3. HTH-type transcriptional repressor purR [NC_019042.1]
4. PTS system, glucose-specific II ABC component [NC_018712.1]
5. hypothetical protein [NC_017567.1]
6. hypothetical protein [NC_018712.1]
7. hypothetical protein [NC_019042.1]
8. truncated hypothetical protein, partial [NC_018712.1]
9. inosine-uridine preferring nucleoside hydrolase [NC_012891.1]
10. flavoprotein NrdI [NC_017567.1]
11. trans-acting regulator [NC_019042.1]
12. bifunctional 2',3'-cyclic nucleotide 2'-phosphodiesterase/3'-nucleotidase precursor protein [NC_017567.1]
13. (p)ppGpp synthetase [NC_017567.1]
14. D-tyrosyl-tRNA deacylase [NC_018712.1]

A. hypothetical protein
B. NTP pyrophosphohydrolase
C. hypothetical protein
D. membrane protein
E. Emm cell surface protein CspZ.1
F. PucR family transcriptional regulator

Figure S3. Schematic representation of the gene arrangement around the *emm* locus in *Streptococcus dysgalactiae* strains. Genes colored in dark green and numbered 1 to 14 are common to more than one *S. dysgalactiae* group. Genes in gray and named A to F are present in either human SDE or SDD but not in *S. dysgalactiae* from horses. Dashed lines below the genes represent variable regions within the group. Numbers above the genes indicate base pairs.

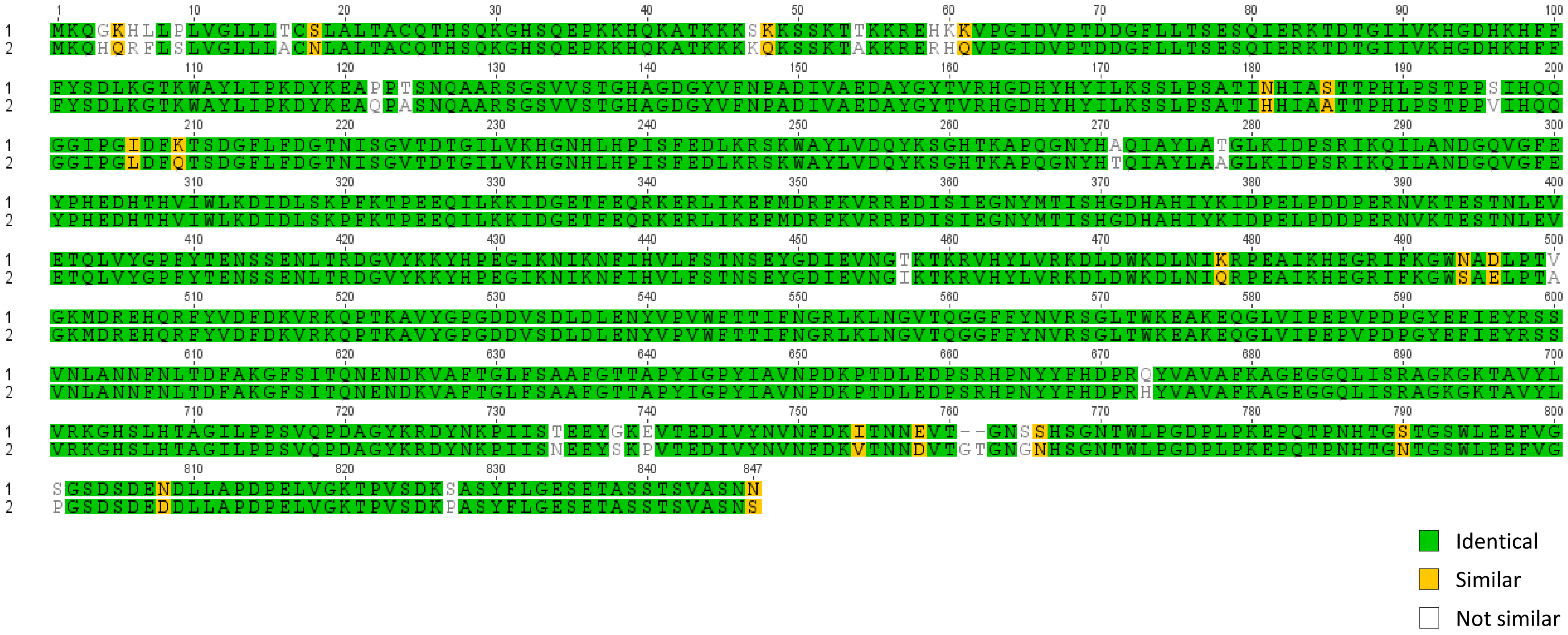

Figure S4. Alignment of the predicted amino acid sequences of putative internalin A-like precursor genes found in (1) horse *S. dysgalactiae* isolate SD24 and (2) *S. equi* subsp. *zooepidemicus* MGCS10565 (protein ID ACG63117.1). Geneious version 8.1 (Biomatters) was used to align the sequences.

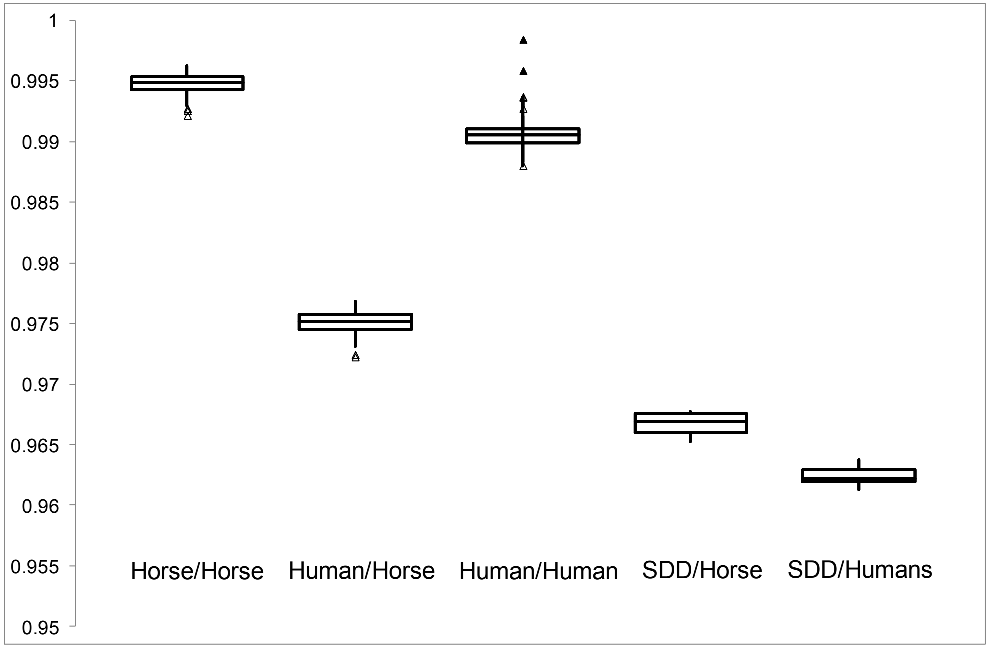

Figure S5. Box plot of the average nucleotide identity (ANIm) between isolates recovered from different hosts.

Table S1. Source, geographic location, Lancefield group and genotypic data of the S. dysgalactiae isolates from horses

Isolate	Source	Country	Year isolation	Lancefield group	MLST ST	*gki*	*gtr*	*murI*	*mutS*	*recP*	*xpt*	*atoB*	*emm* type[a]	*Genome*
VDLUK072	Deep tissues (brain)	USA	2012	C	10	10	4	7	7	12	13	8	*stC7505*	no
UNICAM13	Uterus (uterine swab)	Italy	2008	L	194	22	23	20	23	33	41	24	*stC1*	no
UNICAM23	Uterus (uterine swab)	Italy	2008	L	194	22	23	20	23	33	41	24	*stC1*	yes
VDLUK003	Uterus	USA	1985	L	194	22	23	20	23	33	41	24	NT	no
VDLUK007	Uterus	USA	1985	L	194	22	23	20	23	33	41	24	NT	no
VDLUK020	Uterus	USA	1985	L	194	22	23	20	23	33	41	24	*stC1*	no
VDLUK022	Uterus	USA	1985	L	194	22	23	20	23	33	41	24	NT	no
VDLUK047	Placenta	USA	2011	L	194	22	23	20	23	33	41	24	*stC1*	no
VDLUK050	Fetus (lung)	USA	2011	L	194	22	23	20	23	33	41	24	*stC1*	no
VDLUK056	Fetus (liver)	USA	2012	L	194	22	23	20	23	33	41	24	NT	no
VDLUK054	Fetus (lung)	USA	2012	C	195	23	24	20	23	33	41	25	NT	no
VDLUK035	Uterus	USA	2003	C	196	23	25	20	24	33	42	24	*stC210*	no
VDLUK037	Placenta	USA	2011	C	196	23	25	20	24	33	42	24	*stC210*	no
VDLUK045	Fetus (kidney)	USA	2005	C	196	23	25	20	24	33	42	24	*stC210*	yes
VDLUK051	Placenta	USA	2012	C	196	23	25	20	24	33	42	24	*stC210*	no
VDLUK058	Fetus (lung)	USA	2005	C	196	23	25	20	24	33	42	24	*stC210*	no
VDLUK068	Deep tissues (carpal bone)	USA	2011	C	197	23	25	20	23	33	43	24	*stC210*	no
VDLUK006	Uterus	USA	1985	C	198	23	26	20	23	33	42	8	NT	no
VDLUK014	Uterus	USA	1985	C	198	23	26	20	23	33	42	8	NT	no
VDLUK015	Uterus	USA	1985	C	198	23	26	20	23	33	42	8	NT	no
VDLUK029	Uterus	USA	1986	C	198	23	26	20	23	33	42	8	NT	no
VDLUK030	Uterus	USA	1985	C	198	23	26	20	23	33	42	8	*stC37*	yes
VDLUK060	Fetus (liver)	USA	1986	C	198	23	26	20	23	33	42	8	*stC37*	no
VDLUK064	Deep tissues (bone)	USA	1985	C	198	23	26	20	23	33	42	8	NT	no
VDLUK067	Deep tissues (liver)	USA	2011	C	198	23	26	20	23	33	42	8	NT	no
VDLUK074	Skin and soft tissue (mammary gland)	USA	1985	C	198	23	26	20	23	33	42	8	NT	no
VDLUK075	Skin and soft tissue (abscess)	USA	1985	C	198	23	26	20	23	33	42	8	*stC37*	no
VDLUK079	Skin and soft tissue (skin wound)	USA	1986	C	198	23	26	20	23	33	42	8	NT	no
VDLUK083	Skin and soft tissue (skin abscess)	USA	2011	C	198	23	26	20	23	33	42	8	*stC37*	no
VDLUK094	Respiratory tract (nasal, healthy animal)	USA	2000	C	198	23	26	20	23	33	42	8	NT	no
FUB9198	Unknown	Germany	2004	C	199	23	25	21	23	33	41	8	*stC12*	no
UNICAM02	Vagina (vaginal swab)	Italy	2009	C	199	23	25	21	23	33	41	8	*emm229*	no
UNICAM08	Respiratory tract (tracheal swab)	Italy	2008	C	199	23	25	21	23	33	41	8	*emm229*	no
UNICAM09	Respiratory tract (tracheal swab)	Italy	2008	C	199	23	25	21	23	33	41	8	*emm229*	no
UNICAM10	Respiratory tract (tracheal swab)	Italy	2007	C	199	23	25	21	23	33	41	8	NT	no
UNICAM14	Vagina (vaginal swab)	Italy	2009	C	199	23	25	21	23	33	41	8	*emm229*	no
VDLUK089	Respiratory tract (throat, healthy animal)	USA	2011	C	199	23	25	21	23	33	41	8	NT	no
LMG15901	Respiratory tract (throat)	Sweden	1989	C	200	23	26	20	23	33	43	24	*stC12*	no
VDLUK038	Fetus (kidney)	USA	2011	C	200	23	26	20	23	33	43	24	NT	no
VDLUK088	Respiratory tract (throat, healthy animal)	USA	2011	C	200	23	26	20	23	33	43	24	NT	no
UNICAM18	Skin and soft tissue (abscess)	Italy	2009	C	201	23	25	20	23	33	42	8	*stC11*	yes

Table S1. Source, geographic location, Lancefield group and genotypic data of the S. dysgalactiae isolates from horses

Isolate	Source	Country	Year isolation	Lancefield group	MLST ST	*gki*	*gtr*	*murI*	*mutS*	*recP*	*xpt*	*atoB*	*emm* type[a]	*Genome*
VDLUK036	Uterus	USA	2012	C	201	23	25	20	23	33	42	8	NT	no
VDLUK039	Placenta	USA	2011	C	201	23	25	20	23	33	42	8	NT	no
VDLUK053	Placenta	USA	2012	C	201	23	25	20	23	33	42	8	NT	no
VDLUK057	Fetus (stomach content)	USA	2012	C	201	23	25	20	23	33	42	8	NT	no
UNICAM21	Uterus (uterine swab)	Italy	2007	C	202	23	23	20	23	34	41	24	*stC12*	no
VDLUK048	Fetus (lung)	USA	2011	C	202	23	23	20	23	34	41	24	*stC12*	no
VDLUK073	Deep tissues (liver)	USA	2012	C	202	23	23	20	23	34	41	24	*stC12*	no
VDLUK011	Uterus	USA	1985	C	203	23	25	20	23	33	42	24	*stG5063*	no
VDLUK018	Uterus	USA	1987	C	203	23	25	20	23	33	42	24	*stG5063*	yes
VDLUK046	Fetus (liver)	USA	2011	C	203	23	25	20	23	33	42	24	*stG5063*	no
VDLUK049	Fetus (liver)	USA	2011	C	203	23	25	20	23	33	42	24	*stG5063*	no
VDLUK052	Placenta	USA	2012	C	203	23	25	20	23	33	42	24	*stG5063*	no
VDLUK059	Placenta	USA	1986	C	203	23	25	20	23	33	42	24	*stG5063*	no
VDLUK055	Placenta	USA	2012	C	204	23	23	22	23	34	41	24	*stC12*	no
VDLUK010	Uterus	USA	1986	C	207	23	25	21	23	33	41	24	NT	no
VDLUK076	Skin and soft tissue (laryngeal lymph node abscess)	USA	1985	C	207	23	25	21	23	33	41	24	NT	no
VDLUK027	Uterus	USA	1986	C	208	23	25	20	24	33	42	8	NT	no
VDLUK082	Skin and soft tissue	USA	2011	C	208	23	25	20	24	33	42	8	*stC12*	no
VDLUK084	Skin and soft tissue (submandibular abscess)	USA	2011	C	208	23	25	20	24	33	42	8	*stC12*	yes
VDLUK087	Skin and soft tissue (ear)	USA	2011	C	209	23	25	20	23	33	42	26	NT	no
VDLUK024	Uterus	USA	1985	L	210	23	25	20	23	33	41	24	*stL1*	yes
VDLUK016	Uterus	USA	1985	C	211	23	28	20	23	33	42	24	*stG15*	no
VDLUK032	Uterus	USA	1985	C	211	23	28	20	23	33	42	24	*stG15*	no
VDLUK086	Skin and soft tissue (udder/milk)	USA	1985	C	212	23	24	21	23	36	42	8	*stC17*	yes
VDLUK066	Deep tissues (abdominal fluid)	USA	1985	C	213	23	24	20	23	35	41	*24*	*stC210*	no
VDLUK012	Uterus	USA	1985	L	214	23	29	20	23	33	49	8	NT	no
VDLUK085	Skin and soft tissue (eye)	USA	2011	L	215	23	31	20	23	35	41	24	*stG6*	no
VDLUK008	Uterus	USA	1985	C	216	26	24	20	23	33	41	25	*stC13*	no
VDLUK021	Uterus	USA	1985	C	216	26	24	20	23	33	41	25	*stC13*	no
VDLUK031	Uterus	USA	1985	C	216	26	24	20	23	33	41	25	*stG14*	yes
VDLUK013	Uterus	USA	1985	C	217	27	26	25	23	33	42	24	NT	no
VDLUK033	Uterus	USA	1985	C	217	27	26	25	23	33	42	24	NT	no
VDLUK034	Uterus	USA	1985	C	217	27	26	25	23	33	42	24	NT	no
VDLUK023	Uterus	USA	1985	C	218	28	30	24	24	33	47	8	*stC16*	yes
VDLUK062	Placenta	USA	2011	C	218	28	30	24	24	33	47	8	NT	no
VDLUK071	Deep tissues (gutural pouch)	USA	2012	C	219	29	25	20	23	33	41	8	NT	no
VDLUK026	Uterus	USA	1984	C	220	22	25	20	23	37	41	8	*stC14*	no
VDLUK028	Uterus	USA	1986	C	220	22	25	20	23	37	41	8	*stC14*	no
VDLUK040	Placenta	USA	2011	C	221	23	26	26	23	38	47	24	*stC210*	no
VDLUK041	Fetus (liver)	USA	1985	C	222	23	26	20	23	33	42	28	NT	no
VDLUK042	Fetus (lung)	USA	1985	C	222	23	26	20	23	33	42	28	NT	no

Table S1. Source, geographic location, Lancefield group and genotypic data of the S. dysgalactiae isolates from horses

Isolate	Source	Country	Year isolation	Lancefield group	MLST ST	*gki*	*gtr*	*murI*	*mutS*	*recP*	*xpt*	*atoB*	*emm* type[a]	*Genome*
VDLUK043	Fetus (liver)	USA	1985	C	222	23	26	20	23	33	42	28	NT	no
VDLUK044	Fetus (lung)	USA	1985	C	222	23	26	20	23	33	42	28	NT	no
VDLUK092	Respiratory tract (nasal, healthy animal)	USA	2011	C	223	23	32	20	23	33	42	24	NT	no
UNICAM01	Vagina (vaginal swab)	Italy	2009	C	224	24	25	20	23	35	45	8	*stC14*	yes
UNICAM11	Respiratory tract (nasopharyngeal swab)	Italy	2006	C	225	25	26	23	24	33	46	24	*stC16*	yes
LMG15833	Uterus	Sweden	1989	C	226	23	25	20	24	33	42	26	*stC210*	no
VDLUK004	Uterus	USA	1985	C	226	23	25	20	24	33	42	26	*stC210*	no
VDLUK009	Uterus	USA	1985	C	226	23	25	20	24	33	42	26	*stC210*	yes
VDLUK019	Uterus	USA	1987	C	226	23	25	20	24	33	42	26	*stC210*	no
VDLUK078	Skin and soft tissue (urachus)	USA	1984	C	226	23	25	20	24	33	42	26	*stC210*	no
VDLUK080	Skin and soft tissue (skin abscess)	USA	1986	C	226	23	25	20	24	33	42	26	*stC210*	no
VDLUK081	Skin and soft tissue (udder)	USA	1986	C	226	23	25	20	24	33	42	26	*stC210*	no
UNICAM19	Respiratory tract (broncho-alveolar lavage)	Italy	2009	C	227	23	25	20	24	33	41	8	NT	no
UNICAM20	Uterus (uterine swab)	Italy	2009	C	227	23	25	20	24	33	41	8	*stC8*	no
UNICAM24	Uterus (uterine swab)	Italy	2009	C	227	23	25	20	24	33	41	8	*stC8*	no
UNICAM25	Respiratory tract (tracheal wash)	Italy	2009	C	228	23	25	20	23	33	49	8	*stC11*	no
UNICAM17	Skin and soft tissue (cutaneous fistula)	Italy	2006	C	229	23	27	20	24	33	42	27	*stC210*	no
UNICAM16	Respiratory tract (tracheal wash)	Italy	2009	C	230	23	26	24	25	33	42	24	*stC12*	no
UNICAM03	Uterus (uterine swab)	Italy	2006	C	231	23	26	20	23	33	42	24	*stG2574*	yes
VDLUK093	Respiratory tract (throat, healthy animal)	USA	2011	C	232	31	34	28	6	40	48	8	*stL2764*	no
VDLUK063	Placenta	USA	1986	C	233	30	33	27	26	12	48	29	NT	no
VDLUK091	Respiratory tract (nasal, healthy animal)	USA	1982	C	234	32	33	27	27	39	48	29	NT	no
VDLUK002	Uterus	USA	1981	C	235	31	33	27	28	39	48	30	NT	no
VDLUK017	Uterus	USA	1985	C	236	22	26	20	23	33	41	24	NT	no
VDLUK025	Uterus	USA	1981	C	236	22	26	20	23	33	41	24	NT	no
VDLUK090	Respiratory tract (lung, healthy animal)	USA	2011	C	237	23	25	21	24	33	42	8	*stC12*	no

[a] NT, non-typeable
